# Jejunal Gastrointestinal Stromal Tumor: A Strange Cause of Massive Gastrointestinal Bleeding

**DOI:** 10.7759/cureus.43229

**Published:** 2023-08-09

**Authors:** Daniela Martins, Pedro Costa, Gonçalo Guidi, Pedro Pinheiro, João A Pinto-de-Sousa

**Affiliations:** 1 General Surgery, Centro Hospitalar de Trás-Os-Montes e Alto Douro, Vila Real, PRT

**Keywords:** mesenquimal, lower gastrointestinal bleeding, massive gastrointestinal hemorrhage, jejunal, gastrointestinal stromal tumor (gist)

## Abstract

Jejunal gastrointestinal stromal tumors (GIST) are rare mesenchymal tumors of the gastrointestinal (GI) tract and a rare cause of massive GI bleeding. Due to this rarity and non-specific presentation, diagnosis and treatment may be difficult and often delayed. Urgent surgical intervention is crucial for controlling the source of bleeding and total tumor excision. Herein, we present the case of a 40-year-old male who presented to the emergency room (ER) with features of upper GI bleeding. He referred astheny and black stools, and was pale, sweaty, and tachycardic despite normal blood pressure. Rectal examination revealed melena, and laboratory findings revealed decreased hemoglobin (Hb) and elevated blood urea. Upper endoscopy was normal, and the Hb level dropped again to 6.9 g/dL; therefore, blood transfusion was required during ER observation. For further investigation, the patient underwent an angio-computed tomography scan, which revealed a lesion located in a jejunal loop as the probable bleeding source. Emergency exploratory laparotomy revealed a jejunal loop tumor. Segmental enterectomy containing the tumor was performed and the post-operative period was uneventful. The anatomopathological examination was compatible with low-risk GIST, and the multidisciplinary board agreed that surveillance was the best ongoing treatment. Due to the rarity of jejunal GIST as the cause of massive GI bleeding, diagnosis may be challenging, delaying prompt treatment with bleeding source control. In such cases, surgery may be both lifesaving and curative. Therefore, these tumors should not be forgotten when managing patients with occult GI bleeding with an atypical presentation to prevent delays in treatment and severe outcomes.

## Introduction

Gastrointestinal stromal tumors (GIST) are rare mesenchymal neoplasms of the gastrointestinal (GI) tract and account for 0.4-2 per 100,000 per year. Despite this rarity, these are the most common mesenchymal neoplasms of the GI tract [1,2].

These tumors can arise anywhere in the GI tract and are most frequently found in the stomach (55.6%), small bowel (31.8%), colorectum (6.0%), and esophagus (0.7%). The jejunal location is rare, accounting for 10% of all GISTs [2-4]. Most patients are symptomatic at presentation (69%); 21% are diagnosed incidentally during surgery; and the remaining 10% are incidentally found at autopsy [5].

The diagnosis of small-bowel GIST may be challenging for several reasons, including its relatively low incidence, variable and non-specific symptoms, and vague radiological findings. The symptoms of small-bowel GISTs are non-specific and depend mainly on the tumor size and anatomical location. GI bleeding is the most common symptom (30-40%) [2,5].

In case of GI hemorrhage, prompt source assessment is critical; however, in cases of undiagnosed jejunal GIST this may be challenging. We report the case of an incidentally found jejunal GIST as a rare cause of massive GI bleeding.

## Case presentation

A 40-year-old male, previously healthy, presented to the emergency room (ER), complaining of asthenia, lipothymia, and black stools over the past two days. The patient denied any other symptoms such as nausea, vomiting, abdominal pain, or other symptoms. He had undergone foot orthopedic surgery 10 days earlier, and was medicated with low-molecular-weight heparin (LMWH) and non-steroidal anti-inflammatory agents. The patient was pale, sweaty, and tachycardic (127 beats per minute); however, his blood pressure was normal (117/70 mmHg). An abdominal examination revealed normal findings and a rectal examination revealed melena.

Laboratory findings showed a decrease in hemoglobin (Hb) (11.2 g/dL, with a previous value of 16 g/dL) and a rise in urea 124 mg/dL with no other abnormalities. Serum lactate levels were normal.

**Figure 1:**
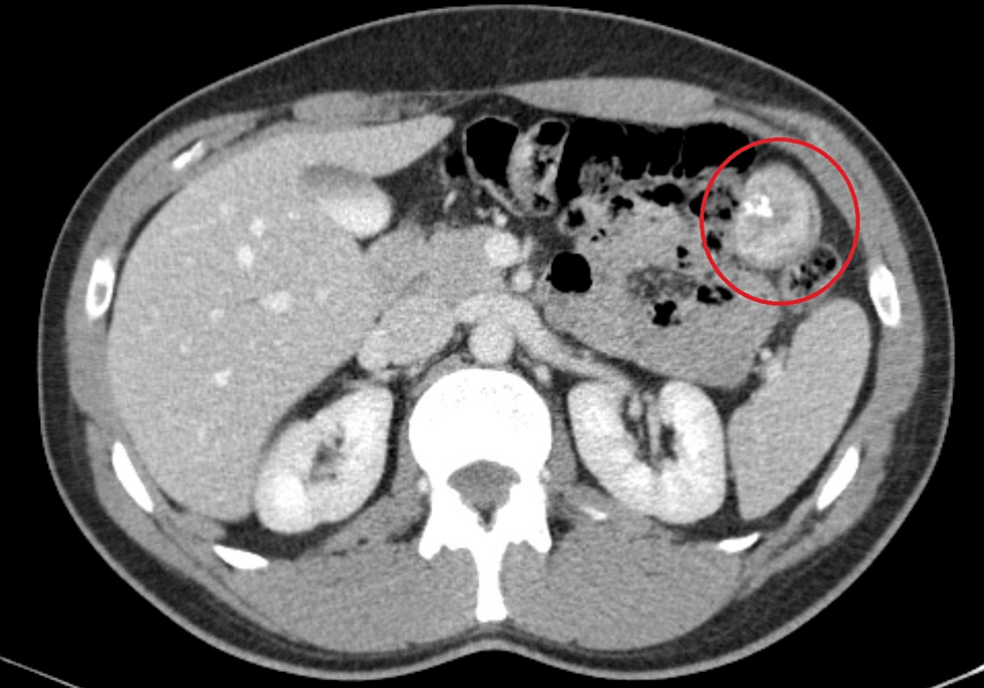
Abdominal CT scan A 3.5cm tumor on small bowel loop, with flush, is presented as a possible hemorrhage source.

The patient was initially responsive to fluid therapy and underwent upper endoscopy with no abnormalities or stigma of hemorrhage detected. Shortly thereafter, he was admitted to the observation room for close surveillance and monitoring, where he remained tachycardic. Repeated laboratory tests identified another Hb drop to 6.90 g/dL requiring two units of red blood cells transfusion which was ineffective. As active bleeding was still on course and endoscopic examination did not reveal a source, the patient underwent an angio-computed tomography (CT) scan that revealed a lesion located in a jejunal loop as the probable source of hemorrhage (Figure 1).

**Figure 2:**
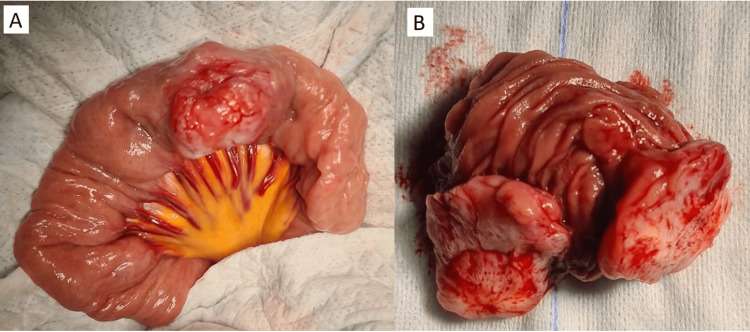
Surgical specimen (A) In situ, showing an ovoid mass in the jejunal loop; (B) after surgical resection and opening.

As the patient did not respond to further transfusions and became unstable, the surgical team decided to perform an emergency exploratory laparotomy. Intraoperatively, a 3.5 cm tumor of hard consistency was found on a jejunal loop nearly 30 cm from the ligament of Treitz with no adhesions to the surrounding structures (Figure 2).

There were no other findings such as free fluid, lymphadenopathies, or metastatic implants. Therefore, a segmental enterectomy containing the tumor was performed followed by a mechanical latero-lateral anastomosis. The postoperative period was uneventful; the patient had no further Hb drop and was discharged on day three.

**Figure 3:**
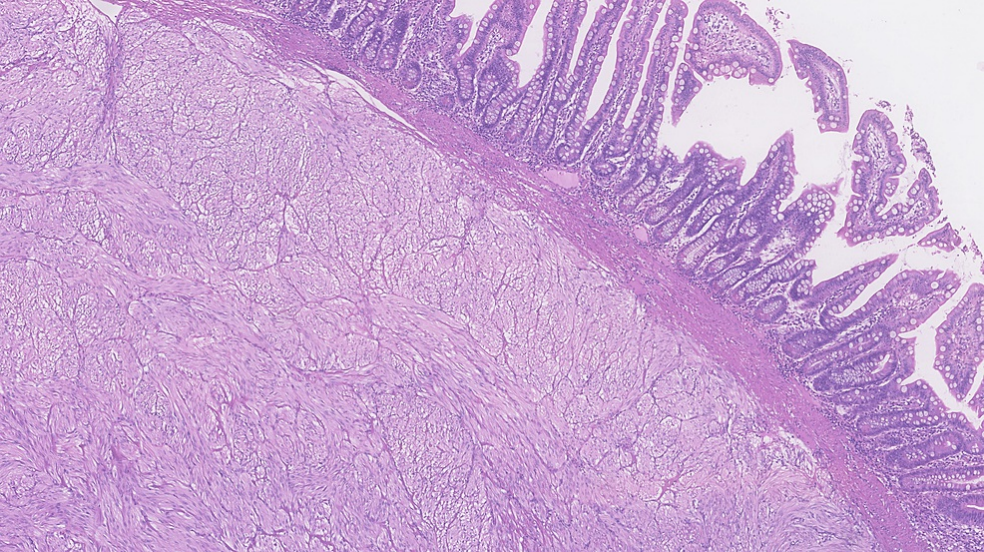
Histopathological findings Area of preserved mucosa and neoplasm occupying the tissue underlying the muscularis mucosa (hematoxylin and eosin staining, magnification ×10).

**Figure 4:**
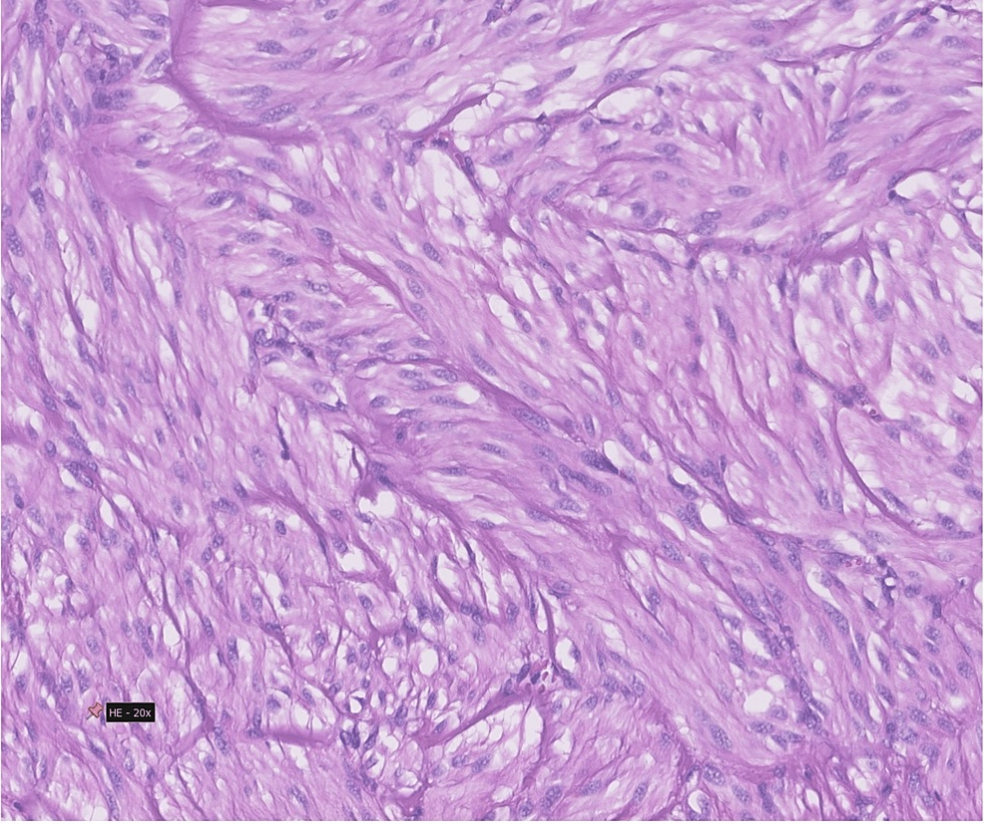
Histopathological findings Spindle cell neoplasm without pleomorphism, necrosis or mitotic figures (hematoxylin and eosin staining, magnification ×20)

Anatomopathological examination revealed a macroscopically ulcerated lesion on the mucosal side of the jejunum, 3.5 cm long, and the mitotic activity was 0 mitosis per 5mm^2^, compatible with a grade 1 stage of pT2 N0 M0 jejunal GIST, according to the American Joint Committee on Cancer Staging Manual (8th edition) Tumor Node Metastasis classification. An R0 resection was achieved (Figures 3, 4) [6].

**Figure 5:**
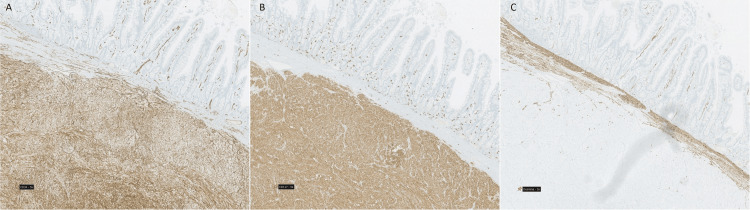
Histopathological findings Immunohistochemical staining for CD34 (A) and CD117 (B) and negativity for desmin (C)

The immunohistochemistry staining demonstrated diffuse expression of CD-117 and CD-34, and negativity for smooth muscle actin (SMA), desmin, and S100 protein (Figure 5). These characteristics were consistent with the diagnosis of low-risk GIST, and the multidisciplinary board decided that surveillance was the best treatment.

## Discussion

Although rare, GISTs are the most common mesenchymal neoplasms of the GI tract [1,2]. GISTs have a slight predominance in males and a median age of 60 years old on presentation [1].

These tumors originate from the interstitial cells of Cajal, a complex cellular network thought to act as pacemaker cells that regulate peristalsis. GISTs can arise anywhere in the GI tract and are more commonly observed in the stomach (55.6%). Less commonly, they may be observed in the small bowel (31.8%), colon and rectum (6.0%), and esophagus (0.7%). There are also sporadic reports of GISTs arising from the mesentery, omentum, and retroperitoneum. Jejunal GISTs are rare (less than 3% of all GI tumors) [2-4,7].

GISTs are symptomatic at the time of diagnosis in 80-90% of cases, and symptoms depend mainly on their size and anatomical location. The most common symptom is GI bleeding (30-40%), followed by abdominal pain (20-50%), and obstruction (10-30%). Massive bleeding with hemodynamic instability is rare and life-threatening, and the hemorrhage may be intra-luminal or, rarely, intra-peritoneal [2,5,8].

The cause of hemorrhage is usually related to rupture of the necrotic or ulcerated component of the tumor. Intraluminal hemorrhage, which is more common, is usually caused by the compression, ischemia, or infiltration of the mucosa by a highly vascularized sub-mucosal tumor. On the other hand, intraperitoneal bleeding due to spontaneous rupture is rare and was reported as 0.11% in a study by Sorour et al. [7]. In our particular case study, hemorrhage might be potentiated by LMWH [7,8].

In a meta-analysis, Fan et al. recently reported that GISTs located in the small intestine with a tumor diameter ≥ 5 cm, a mitotic index ≥ 5/50 high-power fields (HPF), and tumor rupture increase the risk of GI bleeding in patients with GIST [9].

In the present case report, despite the smaller tumor dimension of 3.5 cm and a low mitotic rate, the clinical presentation was a massive GI hemorrhage with hemodynamic instability.

The diagnosis of jejunal GIST may be challenging because of non-specific symptoms, endoscopy inaccessibility, and non-specific features from a CT scan [8].

Upper and lower endoscopies are the first-line investigations for GI bleeding. In the present case, upper endoscopy was normal, and colonoscopy was not performed because there was no time available for bowel preparation in the ER setting. Consequently, the patient underwent an angio-CT scan. Angio-CT plays an important role in cases of GI hemorrhage caused by lesions that are inaccessible to endoscopy. A multiphasic CT scan may locate the bleeding point with 100% accuracy and 85% sensitivity to detect bleeding at a rate of 0.3 mL/min. It may also allow the assessment of tumor characteristics and their relationship to adjacent structures [2,8]. In this patient, the angio-CT scan (RB1) revealed a jejunal mass with active bleeding, and the patient underwent an exploratory laparotomy with prompt jejunal resection.

According to the Current European Society for Medical Oncology-EURACAN Clinical Practice Guidelines for diagnosis, treatment, and follow-up, the usual treatment for localized GISTs is no residual tumor (R0) resection (RB2) with complete surgical excision, without dissection of clinically negative lymph nodes. Therefore, in this case, urgent surgery achieved control of the GI bleeding and was oncologically radical [1].

The definitive diagnosis of GIST is established with pathological examination and immunohistochemistry. The presence of necrosis and mucosal ulceration on pathological examination indicates the cause of GI bleeding. Positive immunochemistry staining for CD-117 is an important hallmark for GIST diagnosis that distinguishes it from other mesenchymal tumors, such as leiomyoma and leiomyosarcoma. Other markers such as CD-34, desmin, SMA, and S100 are rarely positive [5,10].

Important prognostic factors for GISTs are mitotic rate, tumor size, and tumor location. Tumor rupture is an additional adverse prognostic factor that must be recorded. The Armed Forces Institute of Pathology proposed a widely used risk classification to assess the risk of relapse of a localized tumor, which incorporates the three points described above. According to this classification, the patient had a low-risk GIST (absent mitotic rate, less than 5 cm, and located on the small bowel). In such cases, follow-up surveillance without further treatment is recommended [1].

## Conclusions

Jejunal GISTs are extremely rare and may cause massive and life-threatening GI hemorrhage. In these cases, surgery may be lifesaving and curative as a bleeding source controller and if R0 resection is performed.

The angio-CT scan was crucial in determining the bleeding source since it was not visible on endoscopic examination. Therefore, it is critical to keep these tumors in mind when managing patients with occult GI bleeding with an atypical presentation, to prevent delays in treatment and severe outcomes.
